# Marine Fungi as Producers of Benzocoumarins, a New Class of Inhibitors of Glycogen-Synthase-Kinase 3β

**DOI:** 10.3390/md14110200

**Published:** 2016-10-28

**Authors:** Jutta Wiese, Johannes F. Imhoff, Tobias A. M. Gulder, Antje Labes, Rolf Schmaljohann

**Affiliations:** 1 GEOMAR Helmholtz Center for Ocean Research Kiel, Marine Microbiology, Düsternbrooker Weg 20, 24105 Kiel, Germany; jwiese@geomar.de (J.W.); alabes@geomar.de (A.L.); rschmaljohann@geomar.de (R.S.); 2 Biosystems Chemistry, Department of Chemistry and Center for Integrated Protein Science Munich (CISPM), Technical University of Munich, Lichtenbergstraße 4, 85748 Garching, Germany; tobias.gulder@ch.tum.de

**Keywords:** GSK-3β, pannorin, alternariol, alternariol-9-methyl ether, *Aspergillus*, *Botrytinia*, deep-sea

## Abstract

The glycogen-synthase-kinase 3 (GSK-3) is an important target in drug discovery. This enzyme is involved in the signaling pathways of type 2 diabetes, neurological disorders, cancer, and other diseases. Therefore, inhibitors of GSK-3 are promising drug candidates for the treatment of a broad range of diseases. Here we report pannorin (**1**), alternariol (**2**), and alternariol-9-methylether (**3**) to be promising inhibitors of the isoform GSK-3β showing sub-μM IC_50_ values. The in vitro inhibition is in the range of the known highly active GSK-3β inhibitor TDZD-8. Compounds **1**–**3** have a highly oxygenated benzocoumarin core structure in common, which suggests that this may be a new structural feature for efficient GSK-3β inhibition.

## 1. Introduction

Marine environments such as deep sea habitats and macroorganisms provide a great diversity of fungi which are still an underrepresented resource for new drug candidates [1,2]. Natural products produced by marine fungi comprise a large variety of compound classes. Among them are highly active compounds exhibiting a broad range of effects, such as antimicrobial, cytotoxic, or anti-inflammatory activities [3]. As there is a strong need for the discovery of new drugs, for a number of important diseases, the search for bioactive compounds in marine fungi is a timely task. The glycogen synthase-kinase GSK-3β provides a valuable target for drug discovery. GSK-3β is a serine/threonine protein kinase transferring phosphate onto serine and threonine residues. The multifunctional enzyme plays a central role in many signaling processes such as cell proliferation, apoptosis, inflammation, and glycogen metabolism. Therefore, GSK-3β is a therapeutic target for neurodegenerative diseases such as Alzheimer’s disease, Parkinson’s disease, and for diabetes [4,5,6,7]. Furthermore, GSK-3β has been discussed as a potential target for the treatment of cancer [8]. Several GSK-3β inhibitors also from marine origins have been described. Among them are 6-bromoindirubin, dibromocantherelline, and meridianine A, isolated from a marine mollusk, sponge, and tunicate, respectively [9].

According to the World Health Organization (WHO), new drugs are of great relevance for human health worldwide to counteract a number of diseases that are showing increased incidences, in particular diabetes, neurodegenerative disorders, as well as cancer. Diabetes is a major cause of blindness, kidney failure, heart attack, stroke, and lower limb amputation. In the last 25 years, the number of diabetes cases has approximately quadrupled, from 108 million to 422 million. It is estimated that diabetes is the direct cause of 1.5 million deaths per year [10]. The number of people living with dementia worldwide is currently estimated at 35.6 million. This number is expected to double by 2030 [11]. Cancer is also among the leading causes of mortality. In 2012, 8.2 million people died as a result of cancer, and the number of annual cancer cases is expected to rise from 14 million in 2012 to 22 million within the next two decades [12].

This study focuses on three metabolites **1**–**3** which were produced by an *Aspergillus* isolate obtained from the deep sea and by *Botryotinia fuckeliana* obtained from the German Wadden Sea. The compounds share an electron-rich benzocoumarin core, which may be an important new structural element for efficient GSK-3β inhibition and were identified as pannorin (**1**), alternariol (**2**), and alternariol monomethylether (**3**).

## 2. Results

### 2.1. Origin and Classification of the Producer Strains

**Figure 1:**
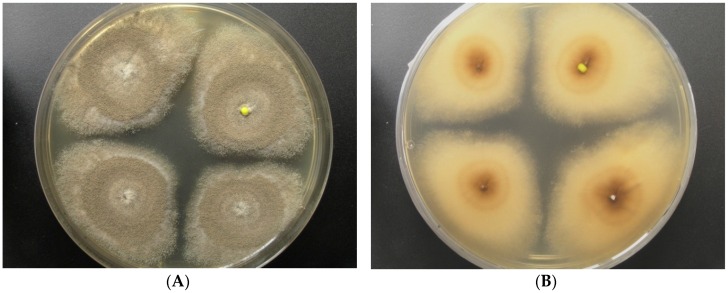
*Aspergillus* sp. LF660, colony grown for 14 days on a modified Wickerham-medium (WSP30) ((**A**): front side, (**B**): back side).

Strain LF660 was isolated in January 2007 from a deep-sea sediment sample (0 to 0.5 cm sediment depth) collected during a cruise of R.V. Meteor 71 leg 2 in December 2006–January 2007 to the Levantine Basin SE of Crete (Mediterranean Sea) in 2769 m water depth. The fungus was identified by the sequence of the ITS1-5.8S rRNA-ITS2 gene fragment, which was 100% identical to the corresponding sequence (accession number FJ80779) of the *Aspergillus sydowii* strain EN50 (Ascomycota, Pezizomycotina, Eurotiomycetes, Eurotiales, Trichocomaceae), and showed only insignificantly less similarity (99.8%) to the closely related *Aspergillus versicolor* strain DY20.1.1 (GenBank accession number LC105698). Therefore, identification of the strain LF660 at the species level is not straightforward and the strain may belong to either of the two species. The strain grew well on, a modified Wickerham-medium (WSP30) producing blue-green colonies of 18 mm diameter within 7 days, which turned to greyish brown during the next 7 days. The back side was colored yellow-brown in the center of the colonies (Figure 1).

**Figure 2:**
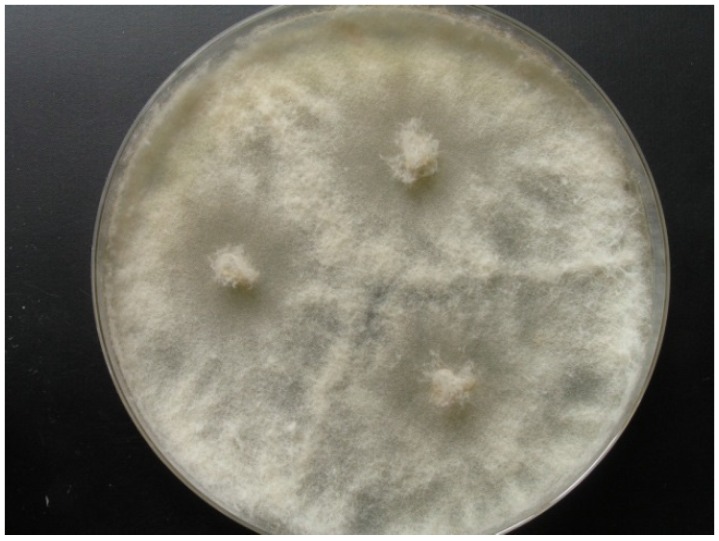
*Botryotinia fuckeliana* KF666, colony grown for 14 days on WSP30 medium.

Strain KF666 was isolated from Wadden Sea water samples of the German Bight collected in March 1996 by K. Schaumann. Sequences of the ITS1-5.8S rRNA-ITS2 gene fragment revealed that this strain affiliated to *Botryotinia fuckeliana* (anamorph *Botrytis cinerea*; Ascomycota, Pezizomycotina, Leotiomycetes, Helotiales, Sclerotiniaceae), with a similarity of 100% to the *B. fuckeliana* strain CLM13701 (GenBank accession number KR055052). Strain KF666 grew rapidly, producing transparent to light grey colonies of up to 75 mm diameter on WSP30 agar within 7 days (Figure 2).

Sequences of the ITS1-5.8S rRNA-ITS2 gene fragments from LF660 and KF666 were submitted to the Genbank database and were assigned to accession numbers KX688043 and KX688044, respectively.

### 2.2. Production of Pannorin *(**1**)* by Aspergillus *sp.* LF660

Strain LF660 is the producer of pannorin (**1**) among other metabolites such as sydonic acid and cladosporin. In order to ensure the sustainable production of larger amounts, we performed experiments to improve the production of **1** and transfer the process from Erlenmeyer flask cultures to controlled stirred tank reactors. Among a number of culture media, the best production was found in WSP30 containing high amounts of glucose and maltose. The replacement of NaCl by artificial sea salt had no effect on the metabolite profile and the production of **1**. In other culture media the production of pannorin (**1**) was negligible (less than 50% compared to WSP30 medium) or absent (data not shown). It was further found that pannorin (**1**) was quantitatively secreted to the culture broth. Such secretion of pannorin was also observed in *Chrysosporium pannorum*, which produced this compound on a potato-starch medium [13].

Culture methods and conditions influence the secondary metabolite production of fungi to a great extent, and often significant differences are observed in surface cultures and submerse cultures as well as in shaken flasks and stirred fermenters (as e.g., for strain KF666 in this study). Therefore, for sustainable large scale production, the reproduction of metabolite production in shaken Erlenmeyer flasks and the transfer of the process into fermenter systems (stirred tank reactors) are the first essential and critical steps. Good growth and production of **1** was achieved in a 10 L fermenter system. The fungus showed improved growth behaviour in a 10 L fermenter system with controlled pH and air saturation (minimum value set to 30%), reaching the exponential phase of growth already after 2 days of cultivation. The elevated oxygen levels in the medium due to supply with air and stirring stimulated hyphal growth. Production of the compound started during the exponential phase with a maximum after 8 days of cultivation. In comparison, a minimum of 17 days was needed to obtain maximum production levels in shaken Erlenmeyer flasks. The production yield was comparable in Erlenmeyer flasks and in the 10 L stirred tank fermenter at 0.4 mg/L. Growth of the fungus and metabolite production in the fermenter stagnated after 10 days and the concentration of **1** remained stable thereafter. The production of pannorin (**1**) in a stirred fermenter system and the significant reduction of the fermentation time is an important first step for a biotechnological production. Based on these experiments, further optimization of the production and scale-up to a technical scale with volumes >200 L can be initiated.

### 2.3. Structural Elucidation

**Figure 3:**
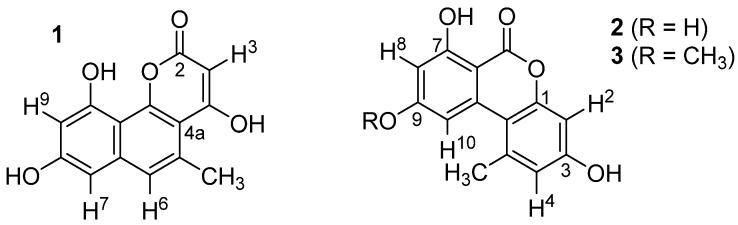
Structures of pannorin (**1**), alternariol (**2**), and alternariol-9-methylether (**3**).

The UV spectra of **1** (*m*/*z* = 258.23) showed maxima at 231, 278, 288, 319, and 363 nm, suggesting the presence of an electron-rich, extended aromatic ring system. The ^1^H NMR spectrum (MeOD-*d*_4_) contained three aromatic protons at 7.18 (H6), 6.60 (H7), and 6.51 (H9) ppm and a sp^2^-bound methyl group at 2.74 (CH_3_) ppm. Small coupling constants between H7 and H9 (^4^*J* = 2.3 Hz) as well as of H6 and the methyl group (^4^*J* = 1.1 Hz) indicated a relative 1,3-position of the corresponding protons. A database search with this information suggested that compound **1** is identical with pannorin (**1**, Figure 3). This was further corroborated by recording NMR spectra of **1** in acetone-*d*_6_, showing slightly shifted signals of the above-mentioned protons (7.23, 6.71, 6.56, 2.78 ppm) and additionally the exchangeable proton H3 (s, 5.58 ppm). Overall, the acquired data were in perfect agreement with that previously reported for pannorin (**1**) [13]. 

The structures of compounds **2** and **3** were evaluated as exemplified for **1**. In these cases, perfect agreement to analytical data found in the literature for alternariol (**2**) [14] and alternariol-9-methylether (**3**) [15] was found (see Table 1).

### 2.4. Biological Activities

Compounds **1**, **2**, and **3** inhibited the activity of the enzyme GSK-3β in a similar range as the positive control TDZD-8, a non-ATP competitive inhibitor belonging to the thiadiazolindiones, which has an IC_50_-value of 0.26 ± 0.03 μM. The highest activity was observed for alternariol (**2**) with an IC_50_ value of 0.13 ± 0.04 μM, followed by alternariol-9-methyl ether (**3**) showing an IC_50_-value of 0.20 ± 0.02 μM. Pannorin (**1**) exhibited an IC_50_-value of 0.35 ± 0.04 μM.

All three compounds had no activity against the causative agents of acne vulgaris (*Propionibacterim acnes*), the plant pathogen causing bacterial leaf spot on tomatoes (*Xanthomonas campestris*), a wheat plant pathogenic fungus (*Septoria tritici*), and the yeast *Candida albicans* at a high test concentration of 100 μM. They poorly inhibited the growth of the Gram-positive test strains *Bacillus subtilis* and *Staphylococcus lentus*. Applying a test concentration of 100 μM, only 54% (**1**), 79% (**2**), and 82% (**3**) inhibition, respectively, were shown for *B. subtilis*, and 88% (**1**), 66% (**2**), and 25% (**3**) inhibition for *S. lentus*, respectively. In addition, the cytotoxic effects against the cancer cell line HepG2 were very slight, because the inhibition was lower than 35% for compound **1**, **2**, and **3**. Taking into consideration the very weak antibacterial and cytotoxic effects of compounds **1**–**3**, their potential as GSK 3β inhibitors should be studied further.

## 3. Discussion

Marine fungi have been found to be an important source for new bioactive metabolites in recent years [1,2,3]. They reveal a wide spectrum of structurally different compounds with a wide range of biological activities. During the search for bioactive fungal metabolites we have identified three benzocoumarins as a new class of efficient inhibitors of GSK-3β, a target with an important role in regulation processes involved in several diseases. 

Pannorin (**1**) has a structure typical of fungal polyketides. It was first reported by Ogawa et al. in 1991 and have been shown to have HMGCoA (3-hydroxy-3-methylglutaryl coenzyme A reductase) inhibiting activity [13]. Recently, pannorin B, a tetracyclic derivative of **1** resulting from an additional acetate unit attached to the 5-methyl group during polyketide biosynthesis and hemi acetale formation of the corresponding keto extension with the phenolic function at C-3 was reported [16]. In addition, this compound and structural analogs thereof were identified during experiments investigating fungal polyketide biosynthesis and pathway engineering [17,18]. 

Alternariol (**2**) is known to form reactive oxygen species (ROS) and to interact with DNA topoisomerase which results in single and double strand DNA breaks followed by reduction in proliferation in mammalian cells, increasing autophagic activity, and induction of senescence. Effects can be often observed in the 5–10 μM range suggesting low acute toxicity [19]. Alternariol-9-methyl ether (**3**) exhibited antibacterial activities with IC_50_ values ranging from 59 nM to 141 nM. Also an anti-nematodal effect was observed with IC_50_ values of 361 nM and 274 nM, respectively [20].

As far as is known, pannorin (**1**), alternariol (**2**), and alternariol-9-methyl ether (**3**) showed only weak toxic activity against microorganisms and human cells. These findings are of great relevance in the evaluation of the compounds as promising drug candidates. Compounds **1**–**3** all have a highly oxygenated, electron-rich benzocoumarin core structure in common. It is tempting to speculate that this could constitute a privileged structural feature for GSK inhibition. The lactone functionality might serve as an electrophilic warhead for a covalent inhibition of this target. Further structure-activity relationship studies and mechanistic investigations with these compounds thus seem to be rewarding. Thus far, known GSK-inhibitors are heterocyclic structures containing one or—in the majority of cases—more nitrogen atoms. Among the 55,000 compounds tested by Baki et al. [21], 149 compounds inhibited GSK >70% at a concentration of 10 μM. The active compounds revealed IC_50_ values between 340 nM and 1000 nM and belonged to aminothiophenes, isothiazoles, thiazolidinones, benzoisothiazoles, and benzothiophenes. As reviewed by Kramer et al. GSK-3β inhibitors are structurally related, e.g., to maleimides (IC_50_ 0.2–304 nM), staurosporines (IC_50_ 0.04–15 nM), indirubines (IC_50_ 2–600 nM), paullones (0.8–6000 nM), pyrimidines (IC_50_ 0.58–2400 nM), furopyrimidines (IC_50_ 5–32 nM), 1,2,5-oxadiazoles (IC_50_ 100–1160 nM), 1,3,4-oxadiazoles (IC_50_ 2.3–65 nM), 1,2,4-oxadiazoles (IC_50_ 350–1130 nM), and thiazoles (IC_50_ 104–560 nM) [22]. Importantly, the benzocoumarins **1**–**3** identified as GSK inhibitors within this study strongly deviate structurally from virtually all known GSK inhibitors, in particular because they are nitrogen-free. They constitute a promising starting point for future in-depth studies on the biological potential and mechanism of GSK inhibitors of this compound class. The IC_50_ values of the benzocoumarins in inhibition of GSK-3β were 130 nM for **2** and 350 nM for **1**.

With the aim of evaluating the potential of the compounds **1**, **2**, and **3** for drug development, further studies have to be carried out that focus on testing purified mammalian GSK-3β, by examining a panel of other kinases as well as the mode of action, e.g., non-ATP competitive or ATP competitive inhibition. 

In terms of the development of promising substances as candidates for drug development from living organisms, it is an important issue to ensure the supply of the compounds. Taking pannorin (1) as an example, it was shown in this study that the production process could be well transferred from culture flasks to a 10 L fermenter. The production of pannorin in a stirred fermenter system and the significant reduction for the production of pannorin is a first important step for a biotechnological production. Based on these experiments, the scale-up to a technical scale with volumes >200 L can be initiated.

## 4. Materials and Methods

### 4.1. Isolation, Cultivation, and Storage of the Producer Strains LF660 and KF666

Both strains were grown on WSP30 agar, a modified Wickerham-medium consisting of 1% glucose, 0.5% peptone, 0.3% yeast extract, 0.3% malt extract, and 3% sodium chloride (pH = 6.8) [23]. The strains were maintained in liquid nitrogen and in the Microbank System at −80 °C (MAST DIAGNOSTIKA, Reinfeld, Germany).

### 4.2. Identification of the Strains LF660 and KF666

For the genetic characterization of the fungi, the ITS1-5.8S rRNA-ITS2 gene fragments were analysed. DNA-extraction and PCR were performed according to Wiese et al. [24]. Closest relatives were identified by sequence comparison with the National Center for Biotechnology Information of the USA (NCBI) Genbank database using BLAST (Basic Local Alignment Search Tool) [25]. Sequence similarity values were determined with the “bl2seq” tool of the NCBI database [26].

### 4.3. Fermentation and Production of Extracts for the Purification of the Compounds ***1***, ***2***, and ***3***

Strain LF660 was inoculated onto agar-plates containing WSP30 medium. After incubation for 35 days at 28 °C, the pre-culture was used for inoculation of 2 L Erlenmeyer flasks containing 750 mL WSP30 medium. The flasks were incubated for 17 days at 28 °C as static cultures in the dark. The mycelium was then separated from the culture medium and 7.5 L fermentation broth was extracted using 6.6 L ethyl acetate. The mycelium was homogenized and extracted using 1.6 L ethanol.

Strain KF666 was cultivated on agar-plates containing WSP30 medium for 21 days at 22 °C prior to the inoculation of 2 L Erlenmeyer flasks containing 750 mL WSP30TM medium (Wickerham medium supplemented with 30 g/L tropic marine salt instead of sodium chloride). Incubation was carried out for 21 days at 28 °C as static and shaken cultures (120 rpm) in the dark. The mycelia of 12 flasks were collected and extracted using 1.8 L ethanol.

Crude extracts of both strains, LF660 and KF666, were evaporated to remove the solvents, re-dissolved in 5 mL methanol, and stored at 4 °C until further use. In order to localise the metabolite, a separation of mycelium and culture broth was performed prior to extraction by filtration (0.45 μm filter membrane filter).

### 4.4. Isolation of the Compounds ***1***, ***2***, and ***3***

Analytical reversed phase HPLC-experiments with diode-array and mass detection were performed using a C_18_ column (Phenomenex Onyx Monolithic C_18_, 100 × 3.00 mm, Phenomenex Inc., Aschaffenburg, Germany) and applying a H_2_O/acetonitrile (ACN) gradient with 0.1% formic acid added to both solvents (gradient: 0 min 5% ACN, 4 min 60% ACN, 6 min 100% ACN; flow 2 mL/min) on a VWR Hitachi Elite LaChrom system (VWR, Darmstadt, Germany) with an L-2450 diode array detector, an L-2130 pump, and an L-2200 autosampler. This HPLC system was coupled to an ESI-ion trap detector with positive ionization (Esquire 4000, Bruker Daltonics, Bremen, Germany) for mass detection.

The preparative HPLC was conducted with a VWR HPLC-UV system (VWR International LaPrep, VWR) equipped with a pump P110, a UV detector P311, a Smartline 3900 autosampler (Knauer, Berlin, Germany), a LABOCOL Vario-2000 fraction collector (LABOMATIC) and a Phenomenex Gemini-NX column (C18, 10 μ, 110 A, 100 × 50 mm, Phenomenex Inc.). A H_2_O/acetonitrile (ACN) gradient with 0.1% formic acid added to both solvents was applied (gradient: 0 min 10% ACN with a flow of 40 mL/min; 0.5 min 10% ACN, 17 min 60% ACN, 22 min 100% ACN, 26 min 10% ACN; flow 100 mL/min). Compound **1** was purified from the extract of the fermentation broth of LF660. Compounds **2** and **3** were obtained from the mycelium extract of KF666. The yields of compound **1**, **2**, and **3** were 3 mg, 13.7 mg, and 6 mg, respectively. 

### 4.5. Structure Elucidation of the Compounds ***1***, ***2***, and ***3***

1H NMR (500 MHz) spectra were measured at 25 °C on a Bruker AVANCE DMX 500 NMR spectrometer with TMS as an internal standard. The signals of the residual solvent protons and the solvent carbons were used as internal references. Masses were acquired using an ESI-ion trap detector with positive ionization (Esquire 4000, Bruker Daltonics).

### 4.6. Production of Pannorin *(**1**)* in a Stirred Tank Reactor

Strain LF660 was cultivated in the 10 L stirred tank reactor system (Biostat, Braun, Melsungen, Germany) with straight propellers using the same type of pre-cultures as for Erlenmeyer flask cultivation. Oxygen concentration, pH, and stirring speed were monitored. The oxygen content in the medium was controlled by adjusting stirrer speed and the aeration rate was set to a minimum of 30% air saturation. Foam formation was stopped by addition of antifoam (Sigma, Taufkirchen, Germany). After cultivation, cells were separated from the culture broth by means of centrifugation. For the 1 L and 10 L scale, culture supernatant and cells were extracted by the addition of 2 volumes ethyl acetate. The organic solvent was separated and concentrated to dryness under reduced pressure.

### 4.7. Biological Activities Assays

The inhibition of GSK-3β by compounds **1**, **2**, and **3** was studied using the luminescent assay modified from Baki et al. [21]. The assay is based on the phosphorylation of a peptide by the enzyme with ATP as phosphate donor. ATP, luciferin, and oxygen react to oxyluciferin, which is measured as a luminescence signal. The more the enzyme is inhibited, the stronger the luminescence signals are because of high ATP levels. 20 μL substrate solution (2 μM ATP, 40 μM peptide HSSPHQ-pSer-EDEEE (BIOSYNTAN, Berlin, Germany)) and 10 μL enzyme solution (12 mU GSK-3β (N-terminal His6-tagged recombinant human GSK3β with an H350L mutation, EMD Millipore, Billerica, MA, USA)) were incubated for 60 min at 25 °C. Then 40 μL Kinase-Glo reagent (Kinase-Glo Luminescent Assay Kit, Promega, Darmstadt, Germany) was added. After centrifugation and incubation for 10 min at 25 °C, the luminescence was measured. TDZD-8, 4-Benzyl-2-methyl-1,2,4-thiadiazolidine-3,5-dione, (Merck, Darmstadt, Germany), was used as the positive control. The antimicrobial activities of compounds **1**, **2**, and **3** against the bacterium *Bacillus subtilis* (DSM 347) and the human pathogenic yeast *Candida albicans* (DSM 1386) were determined according to Ohlendorf et al. [27]. The determination of inhibitory activities against *Xanthomonas campestris*, *Septoria tritici*, and *Propionibacterium acnes*, as well as the cytotoxic activity against the cell line HepG3 was carried out as described by Schneemann et al. [28].

## 5. Conclusions

The systematic search of substances from marine fungi, their structure elucidation, and biological activities have established quite a number of new substances in recent years [1,2,3]. In the present study, for the first time, three benzocoumarins were found to express significant inhibition of GSK-3β. Pannorin (**1**), alternariol (**2**), and alternariol-9-methyl ether (**3**) were shown to be new effective GSK-3β inhibitors sharing a highly oxygenated benzocoumarin core structure and exhibiting significant inhibitory activity with an IC_50_ value range of 130 nM–350 nM. This inhibition and the apparent lack of significant antimicrobial and cytotoxic activities are appearing for further study of other benzocoumarins in the context of GSK inhibition and their possible applications in medical treatments.

## Figures and Tables

**Table 1:** Comparison of ^1^H NMR data of isolated compounds **2** (in MeOH-*d*_4_) and **3** (in DMSO-*d*_6_) with literature data recorded in the identical solvents [14,15].

Position	Alternariol (2)		Alternariol-9-methylether (3)	
	Isolated ^a^	Literature ^a^	Isolated ^a^	Literature ^b^
H-2	6.55, d (2.6)	6.55, d (2.5)	6.72, d (2.6)	6.72, d (2.3)
H-4	6.64, d (2.6)	6.65, d (2.5)	6.64, d (2.6)	6.64, d (2.4)
H-8	6.31, d (2.1)	6.32, d (2.0)	6.61, d (2.2)	6.61, d (2.3)
H-10	7.20, d (2.1)	7.20, d (2.0)	7.23, d (2.2)	7.21, d (2.3)
5-CH3	2.70, s	2.71, s	2.73, s	2.73, s
O-CH3	-	-	3.90, s	3.90, s
3-OH	-	-	10.38, brs	10.36, brs
7-OH	-	-	11.82, brs	11.81, brs

^a^ recorded at 500 MHz; ^b^ recorded at 400 MHz.
